# The Association Between Polycystic Ovary Syndrome and Vitamin D Deficiency in Infertile Women: A Case‑Control Study [Letter]

**DOI:** 10.1002/hsr2.71948

**Published:** 2026-02-28

**Authors:** Hongjing Chen, Haobo Chen

**Affiliations:** ^1^ Wenzhou Hospital of Integrated Traditional Chinese and Western Medicine Affiliated to Zhejiang Chinese Medical University Wenzhou China

Dear Editors,

We read with great interest the article titled “The Association Between Polycystic Ovary Syndrome and Vitamin D Deficiency in Infertile Women: A Case‑Control Study [1].” Polycystic ovary syndrome (PCOS) is characterized primarily by ovulatory dysfunction, hyperandrogenism, and polycystic ovarian morphology and constitutes a significant contributor to female infertility. Vitamin D deficiency is prevalent among women, and the authors offer novel insights by examining the association between vitamin D status and infertility in patients with PCOS. We would like to raise several points for clarification and further discussion that may help interpret the findings and guide future research.

First, the diagnostic criteria for infertility were not specified. This study enrolled women with PCOS and infertility, and reported the distribution of primary and secondary infertility. However, the diagnostic criteria for infertility were not clearly defined. According to the 2025 WHO guideline, infertility is defined as the failure to achieve pregnancy after 12 months or more of regular unprotected intercourse [2]. Clarifying this definition would improve the interpretability and comparability of the study population.

Second, although the authors reported a post hoc power analysis, the achieved sample size (66 per group) was smaller than the initially planned sample size. It would be helpful to further discuss how this deviation from the original sampling plan might influence the stability and generalizability of the estimated effect sizes, particularly in a multivariable model adjusting for several confounders.

Third, insulin resistance is a key pathophysiological feature of PCOS and is closely linked to both vitamin D status and metabolic outcomes [3]. While the authors appropriately acknowledged the absence of direct insulin resistance markers, such as HOMA‐IR, it would be informative to clarify whether any proxy indicators (e.g., history of diabetes or use of insulin‐sensitizing medications) were assessed or excluded [4, 5]. Explicitly addressing this point would help readers better understand the potential impact of residual confounding.

In summary, this study provides valuable local data on vitamin D deficiency in infertile women with PCOS. Clarification of infertility definitions, further discussion of sample size considerations, and a more detailed description of insulin‐related confounding would further strengthen the methodological transparency and clinical interpretability of the findings.

## Author Contributions

**Hongjing Chen:** conceptualization, writing – original draft, writing – review and editing. **Haobo Chen:** writing – review and editing.

## Funding

The authors received no specific funding for this work.

## Conflicts of Interest

The authors declare no conflicts of interest.

## Data Availability

Data sharing is not applicable to this article, as no new data were created or analyzed in this study.
